# Case report: Applied behavior analysis in a case of anomic aphasia in post-acute myocardial infarction with cardiac arrest and brain hypoxia: results of tact-training

**DOI:** 10.3389/fpsyg.2024.1407399

**Published:** 2024-06-27

**Authors:** Valentina Catania, Guido D’Angelo, Simonetta Panerai, Bartolo Lanuzza, Raffaele Ferri

**Affiliations:** ^1^Oasi Reseach Institute - IRCCS, Troina, Italy; ^2^Disability and Health Integrated Program, Local Health Unit, Bologna, Italy; ^3^Cooperativa Dalla Luna, Bari, Italy

**Keywords:** aphasia, anomic aphasia, stroke, ABA, applied verbal behavior, verbal operants

## Abstract

**Purpose:**

Applied Behavior Analysis (ABA) tact-training was provided to an adult with post-stroke anomic aphasia, with the main purposes to improve naming of pictures, with a possible generalization to another different setting, through telehealth sessions.

**Method:**

The Multiple probe experimental design across behaviors was used. Two sets of stimuli were used (SET 1 and SET 2), including 60 laminated photos, belonging to three different categories for each set. Procedure included the baseline, the intervention phases (face-to-face and telehealth sessions), and the follow-up (1 month after the end of a tact training).

**Results:**

For both, SET 1 and SET 2, the mastery criterion (80% correct stimulus tacts, for three consecutive times, simultaneously for all categories) was achieved. No increased percentage of correct picture tacts was found for untrained items. At follow-up, the patient provided 70 to 100% correct responses. For both SET 1 and SET 2, telehealth did not modify the correct response trends.

**Conclusion:**

The results of our study seem to suggest that specific tact-training procedures might be successfully carried out in adult and elderly people with post-stroke aphasia. It also appears necessary to arrange protocols providing telehealth sessions, with benefits for both families and the health system.

## 1 Introduction

Cerebral hypoxia, common in cardiac arrest (Wagner et al., 2020), can lead to brain lesions, potentially impairing brain function. Approximately 20–50% of cardiac arrest survivors, and those who survive myocardial infarction, may experience a decline in global cognition and long-term cognitive impairments, including language difficulties such as anomia (Middelkamp et al., 2007; Polanowska et al., 2014; Cronberg et al., 2020; Wagner et al., 2020; Johansen et al., 2023). While cognitive function may improve within the first year post-event, long-term deficits persist, impacting independence and quality of life (Middelkamp et al., 2007; Polanowska et al., 2014), highlighting the necessity for specialized cognitive rehabilitation.

Aphasia, resulting from brain lesions, affects verbal expression and comprehension components, classified into non-fluent and fluent conditions (Sinanović et al., 2011; Sheppard and Sebastian, 2021). Anomic aphasia, a mild fluent type, involves word retrieval difficulties while maintaining comprehension and fluency (Lorwatanapongsa, 2005; Sheppard and Sebastian, 2021), often attributed to left hemisphere lesions, primarily in the parietal–temporal junction (Whatmough et al., 2002).

Moreover, functional communication, as described by van der Meulen et al. (2010), referring to the practical use of language and communication skills in real-life situations to achieve specific goals and meet daily communication needs is also impaired in aphasia.

Although no universally effective treatment exists for aphasia (Fridriksson and Hillis, 2021; Vitti and Hillis, 2021), semantic-feature-based treatments (SFTs) seem to be effective in aphasia therapy. Bontemps et al. (2024) found that combining executive function training with SFA improved naming and discourse in chronic aphasia. Scimeca et al. (2024) demonstrated significant naming improvements in bilingual aphasia, influenced by language treatment and baseline severity. Hameau et al. (2019) showed that semantic neighborhood density affects naming accuracy in aphasia, highlighting the role of semantic context in word production. To our knowledge, Applied Behavior Analysis (ABA) has not been explored for anomic aphasia. ABA focuses on understanding and shaping human behavior by manipulating environmental variables (Cooper et al., 2020). Applied Verbal Behavior (AVB), a subset of ABA, emphasizes verbal function rather than form (Cooper et al., 2020).

A recent scoping review concluded that, although telehealth methods seem to be valid for the treatment of aphasia, further research is required to explore the variety of aphasia assessment and intervention protocols that can be delivered through telehealth (Teti et al., 2023).

This report presents ABA tact training for anomic aphasia rehabilitation in a patient with mild Neurocognitive Disorder (m-NCD; American Psychiatric Association, 2013) 1 year post-acute myocardial infarction with cardiac arrest and brain hypoxia, the first such study. Following Lorwatanapongsa’s (2005) treatment aims for anomic aphasia, our objectives were to improve picture naming and sorting, generalize gains through telehealth sessions, extend to untrained pictures, and assess retention after 1 month.

## 2 Method

### 2.1 Study design

The Multiple probe experimental design across behaviors was used (Horner and Baer, 1978).

### 2.2 Participant

SL is a 57-year-old Sicilian man. In April 2020 he suffered an acute myocardial infarction with cardiac arrest and brain hypoxia. He underwent resuscitation maneuvers for 40 min, then coronary revascularization surgery, during which a new cardiac arrest occurred for a few minutes. A stent was inserted. Transferred to the Intensive Care Unit, the patient regained consciousness after about 15 days, showing good hemodynamic compensation. He manifested marked memory and orientation deficits (he did not remember his name, his age, he did not recognize his family members), as well as psychomotor agitation, for which he was pharmacologically sedated. He did not show motor deficits. After discharge, he gradually recovered self-help skills at home, but presented language impairments (especially anomia). After 1 year from the cardiac arrest, he underwent a medical check-up at our Institute. During this year no specific rehabilitation treatments were implemented. A diagnosis of cerebral vascular disease, brain hypotrophy, anomic aphasia and m-NCD was made by our multidisciplinary team (including a geriatrician, a neurologist, an otolaryngologist, a speech pathology doctor, a speech therapist, and a neuropsychologist). At the end of the hospitalization, speech and cognitive rehabilitation treatments were recommended. Upon admission to the Neuro-rehabilitation Unit of our Institute, the patient was on ticagrelor, simvastatin, acetylsalicidic acid, bisoprolol, ramipril, and pantoprazole. Brain magnetic resonance imaging showed hyperintensity of the white matter in the right temporo-occipital area (near the temporal horn of the lateral ventricle), dilatation of the ventricular system and pericerebral and pericerebellar brain spaces. The electrocardiogram showed complete right bundle branch block and left posterior hemiblock, and frequent polymorphic ventricular ectopic beats.

After obtaining the written informed consent by the patient, a neuropsychological battery – Italian adaptation of the Uniform Data Set Neuropsychological Test Battery (Conca et al., 2022) – was administered. This battery covers several cognitive domains including attention, processing speed, executive function, episodic memory, and language. The battery consists of standardized tests designed to assess these cognitive functions comprehensively and reliably in clinical and research settings. A cognitive profile characterized by normal visual reasoning, verbal and visual–spatial span, and visual selective attention was found; the basic Activities of Daily Living were maintained. On the contrary, mild deficits were found in long-term verbal and visual recall, executive functioning, and Instrumental Activities of Daily Living. The language assessment, carried out by means of the Italian version of the Aachener Aphasia Battery (Luzzatti et al., 1996), showed moderate deficit in naming, and mild deficit in repetition, comprehension, and written language (both reading and writing).

### 2.3 Setting

The tact training was administered by a Board-Certified Behavior Analyst (BCBA) neuropsychologist, supervised by a senior BCBA. It included 58 face-to-face and 25 telehealth sessions, one follow-up per category and three generalizations per category (see below). The face-to-face sessions were carried out in a well-lit room, about 4 × 3 m, equipped with a desk, two chairs, and a bookcase; trainer and patient sat facing each other. Sessions lasted about 50 min. After discharge, treatment continued in telehealth (starting from the 59^th^ session), by means of *WhatsApp* video-calls. Co-therapist was the patient’s wife, who implemented the procedure according to the instructions of the trainer, simultaneously connected online.

### 2.4 Materials

Two sets of stimuli, SET 1 and SET 2, were used in the face-to-face sessions, each including 60 laminated colored photos 10×10 cm, divided into three categories. SET 1 included *animals-fruits-vehicles*; SET 2 *vegetables-foods-clothes*. In session 75 of SET 2, since no substantial improvements were observed for the *food category*, this was replaced with the *household items* category. The hypothesis behind this change was that the two categories - *vegetables and foods* - were semantically too similar. During the telehealth phase, stimuli were instead shown on a screen (photos on white background). Stimuli in each category were balanced according to the frequency of use and word length.

## 3 Procedure

The steps of the procedure are detailed in the following paragraphs.

### 3.1 Baseline

For each category, baseline data were recorded, regarding stimulus tact, category tact and sorting, three data points for SET 1, and five for SET 2. For *household items*, which replaced the *food* category, three data points were collected. Half of the stimuli used for baseline were proposed during tact training and follow-up; the other 50% was retested at the end of tact training, in order to verify generalization to untrained pictures.

### 3.2 Intervention phases

#### 3.2.1 Face-to-face tact training

Fifty-eight face-to-face sessions (two sessions a day for 5 days a week, from Monday to Friday) were carried out. During tact training, 10 stimuli for both SET 1 and SET 2 categories were used. In each session, 30 pictures were shown to the patient in a random sequence, and he was required to name each picture. The patient was asked to superimpose each named picture on one of three images placed in front of him, each representative of one specific category, and then to label categories. The three images were randomly selected at each session from a group of nine images (three per category). Each correct response was followed by a positive social reinforcement associated with a token, which was placed inside a box. At the end of each session, tokens were counted and compared with those obtained in the previous session. This reinforcement motivated the patient, who wanted to achieve an ever-higher score. Whenever the patient did not respond within 5 sec, an echoic prompt was provided and the patient was requested to immediately practice the correct response (Cooper et al., 2020); when the patient himself required additional time to provide the response, the echoic prompt was delayed by up to 30 s. If the patient responded incorrectly, a correction procedure – echo feedback plus practice of the correct response (McGhan and Lerman, 2013; Carroll et al., 2018) – was applied. The mastery criterion was set at 80% of correct stimulus tacts, on all SET categories simultaneously, for three consecutive times; once the criterion was reached, the next stages of treatment were carried out.

#### 3.2.2 Telehealth tact training

Five weekly sessions were carried out, one per day, from Monday to Friday. The same procedure described above was applied. Through a video-call, the trainer framed each picture, so that the patient could see it clearly, and then named it and its relevant category. After naming the category, the patient showed it to the trainer by taking one of the three images (one per category) which he had personally placed on the table before the session. For each correct response, a positive social reinforcement and a token were provided to the patient (as the session was remotely carried out, the token consisted in ticking a relevant form delivered to the patient at discharge). At the end of each session the sum of the tokens was compared with that obtained in the previous session. The use of the echoic prompt, the correction procedure and the mastery criterion were the same as described above.

#### 3.2.3 Generalization to untrained pictures

On both SET 1 and SET 2, when the mastery criterion was met, the baseline was retested on the 30 untrained stimuli.

#### 3.2.4 Follow-Up

For both SET 1 and SET 2, 1 month after the conclusion of the tact training, the retainment of the re-acquired tacts was tested during a telehealth session.

### 3.3 Scoring and data analysis

Counting was used for stimulus tacts, category tacts, and sorting. Percentages were then calculated.

### 3.4 Procedural integrity: interobserver agreement calculation

A second independent observer collected data on the participant performance in 30% of sessions. The IOA was calculated by dividing the number of agreements by the number of disagreements and multiplying the result by 100. The IOA average was 98.7% (range 97.3–100%).

## 4 Results

**Figure 1:**
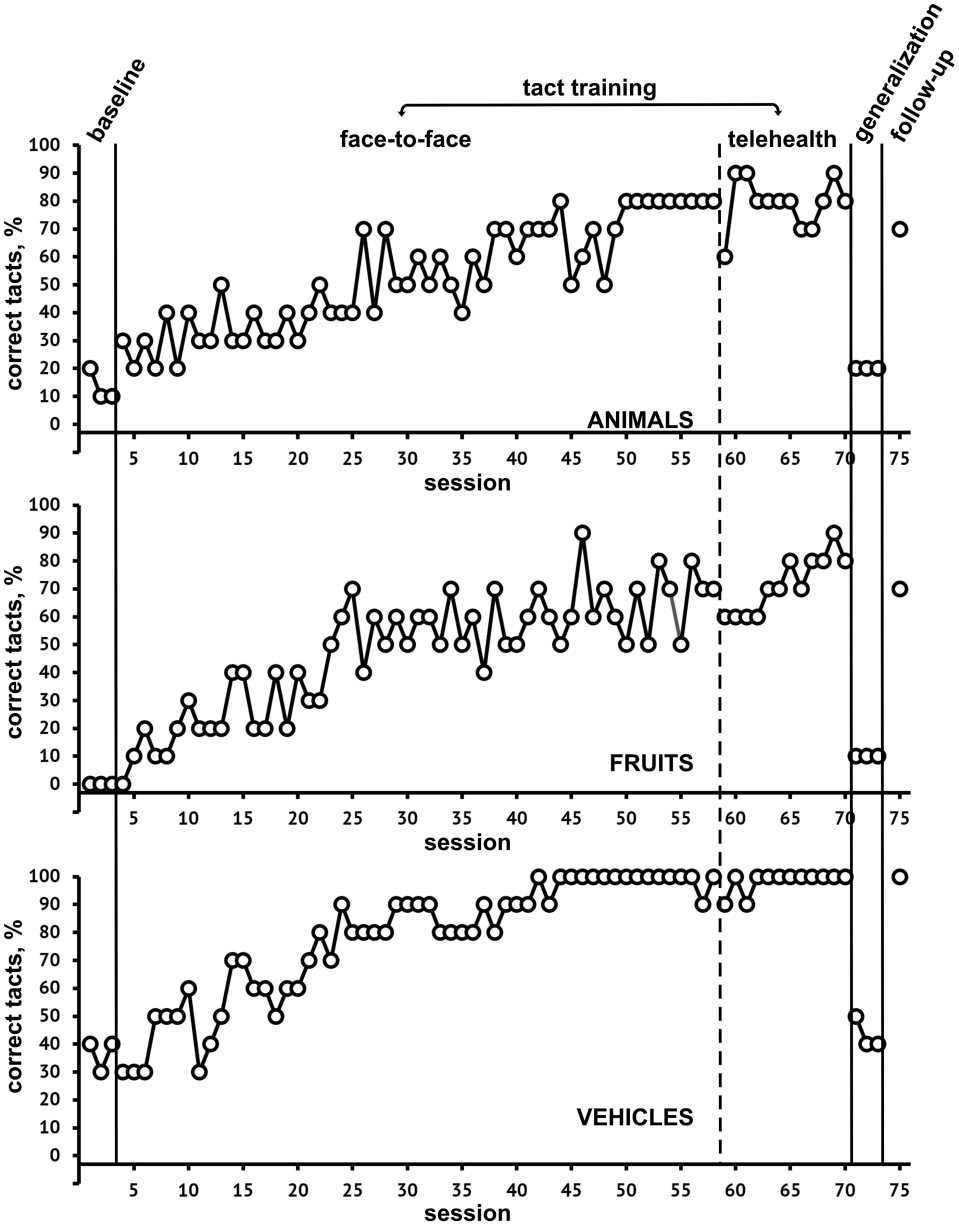
Percentage of correct tact responses at baseline, during both face-to-face and telehealth tact training, generalization, and follow-up for SET-1, categories Animals, Fruits, and Vehicles.

**Figure 2:**
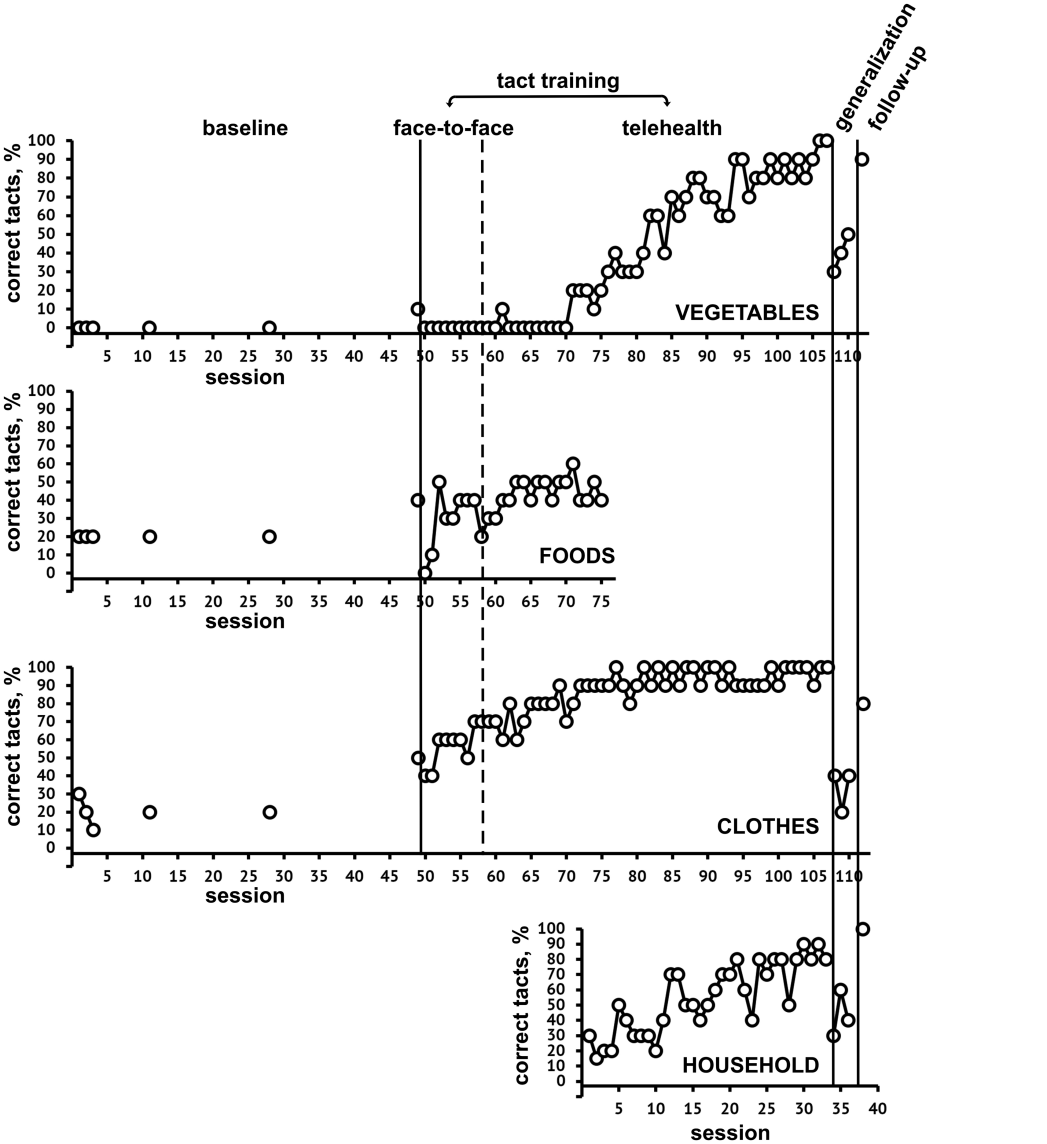
Percentage of correct tact responses at baseline, during both face-to-face and telehealth tact training, generalization, and follow-up for SET-1, categories Vegetables, Foods, Clothes, and Households.

Results are shown in Figure 1 (SET 1) and Figure 2 (SET 2). As for SET 1 (*animals-fruits-vehicles*), the mastery criterion (80% correct stimulus tacts for three consecutive times, simultaneously for all categories) was achieved in session 70, during the telehealth phase. Correct tacts of pictures in each category gradually increased over time, especially *vehicles*, for which the 80% correct responses was achieved at the 27^th^ session. Category tacts and sorting quickly reached 100% correct responses, but also at baseline correct responses ranged from 80 to 100%. At the retest time of untrained stimuli (second baseline), no increase in percentages of correct picture tacts was found; instead, category tacts and sorting showed 100% correct responses. At follow-up the patient provided 70% correct responses in *animal and fruit* pictures, and 100% in *vehicles*. Category tacts and sorting showed 100% correct responses at follow-up, too.

As for SET 2 (*vegetables, clothes, household*), the mastery criterion was achieved at session 110. The graph shows a gradual increase over time in percentages of *clothes* pictures correctly named, for which the mastery criterion was achieved in session 65. *Vegetable and food* pictures did not show an appreciable increase in correct responses up to the 75^th^ session. For this reason, the *food* category was replaced by the *household* one, and after this change a gradual increase over time of both *vegetables* and *household* correct picture tacts was shown; also category tacts and sorting quickly reached 100% correct responses. At the untrained stimuli retest, no increase in correct responses was found. At follow-up, 70 to 90% correct picture tacts were achieved, 100% in category tacts and sorting.

For both SET 1 and SET 2, the transition to telehealth intervention did not lead to significant modifications in the trends of correct responses.

## 5 Discussion

Aphasia profoundly affects patients’ daily lives, along with their families and friends, leading to changes in social roles, job loss, and social isolation (Boden-Albala et al., 2005; Baker et al., 2018; Berg et al., 2020). Two main behavioral interventions for post-stroke aphasia are identified: Impairment-based approaches, focusing on specific language tasks improvement, and Functional communication approaches, emphasizing context and multimodality of communication (Doedens and Meteyard, 2022). To our knowledge this is the first study in which ABA procedures were implemented in a case of aphasia associated with cognitive disorders. ABA, emphasizing functional links between behavior and the environment, breaks down expressive language into functional sub-units (verbal operants) (Skinner, 1957). While past literature has overlooked ABA-based treatments for acquired language disorders (Heinicke and Carr, 2014; Becker and Baltazar, 2022), this study aims to contribute to this field.

Regarding our study objectives, we focus on enhancing correct picture tacts, especially in different category sets. Sorting and category tact improvements were less significant, except for household categories, showing immediate increases. Baseline correct responses were already high for SET 1 and clothes of SET 2, while instability in vegetable category responses was observed until session 75, possibly due to interference with the food category.

Results showed a functional relationship between tact training and increased correct picture tacts, meeting the mastery criterion for trained stimuli. This suggests that specific tact training procedures can effectively rehabilitate post-stroke anomic aphasia, expanding rehabilitation options beyond traditional treatments.

While ABA-based treatments are uncommon in Italy for adult neurocognitive disorders, our patient’s outcomes suggest their potential inclusion in rehabilitation protocols, potentially improving outcomes and reducing healthcare costs. Telehealth sessions post-discharge showed similar trends to face-to-face sessions, indicating their potential for cost-effective remote rehabilitation. This can be considered a positive result because telehealth seems to provide important social and economic benefits for patients and their families, as well as the health system (Jennett et al., 2003).

Generalization to untrained pictures did not show improvement, consistent with previous studies suggesting limited generalization in aphasia treatment effects (Quique et al., 2019; Scholl et al., 2021). The observed retention of correct picture tacts 1 month post-training suggests satisfactory outcomes, though further studies are warranted to explore influencing factors and long-term maintenance of gains.

Strengths of our study include being the first to apply ABA tact training in acquired anomic aphasia, demonstrating its potential effectiveness in adults with neurocognitive disorders. Telehealth sessions’ effectiveness post-discharge indicates their potential for cost-effective remote rehabilitation.

Limitations include single-participant application and the need for further studies across different aphasia types and neurodegenerative disorders. Careful category selection is crucial to avoid interference effects. Despite achieving mastery criteria for trained stimulus sets, our study found no significant increase in correct responses for untrained items. Enhancing the generalization of learned skills to a broader range of stimuli could strengthen the practical applicability and effectiveness of the intervention. Generalization to real objects warrants further investigation to enhance the social validity of rehabilitation interventions. The study’s sample size and diversity could be improved to enhance the generalizability of findings to a broader population of individuals with post-stroke aphasia.

### 5.1 Clinical implication and future studies

Our study supports the efficacy of ABA procedures in speech rehabilitation of patients with stroke-related aphasia. ABA procedures might therefore be more widespread in the rehabilitation of adult and elderly patients with post-stroke cognitive and speech impairments. The effects of ABA trainings might also be tested in patients with cognitive and speech deficits due to neurodegenerative dementia conditions.

Essential for future studies it would also be to evaluate the effects of transfer trainings from one verbal operant to another, e.g., from tact to mand, or intraverbal, in order to open up the possibility for the patient to use the relearned words with different functions, making speech more natural. Generalization of naming could potentially be measured with a tool measuring functional communication like the Scenario Test (Van der Meulen et al., 2010). Our findings also seem to suggest an extension of telehealth for the speech therapy of patients with post-stroke aphasia. For patients, telehealth sessions might be integrated with face-to-face rehabilitation sessions through specific protocols; conversely, caregivers might benefit from telehealth sessions to learn how to apply the behavioral procedures at home. Future research could also delve deeper into the efficacy of telehealth-delivered interventions, including factors such as patient engagement, session adherence, and technological considerations.

## Data availability statement

The datasets presented in this article are not readily available because privacy and ethical aspects. Requests to access the datasets should be directed to cataniavalentina@hotmail.it.

## Ethics statement

The studies involving humans were approved by COMITATO ETICO IRCCS SICILIA-OASI MARIA SS. The studies were conducted in accordance with the local legislation and institutional requirements. The participants provided their written informed consent to participate in this study. Written informed consent was obtained from the individual(s) for the publication of any potentially identifiable images or data included in this article.

## Author contributions

VC: Conceptualization, Data curation, Investigation, Methodology, Supervision, Validation, Writing – original draft, Writing – review & editing. GD’A: Conceptualization, Formal analysis, Supervision, Validation, Writing – review & editing. SP: Conceptualization, Data curation, Supervision, Writing – original draft, Writing – review & editing. BL: Data curation, Writing – review & editing. RF: Data curation, Funding acquisition, Visualization, Writing – review & editing.
